# Hematopoietic transcription factors and differential cofactor binding regulate *PRKACB* isoform expression

**DOI:** 10.18632/oncotarget.17386

**Published:** 2017-04-24

**Authors:** Olga N. Kuvardina, Stefanie Herkt, Annekarin Meyer, Lucas Schneider, Jasmin Yillah, Nicole Kohrs, Halvard Bonig, Erhard Seifried, Carsten Müller-Tidow, Jörn Lausen

**Affiliations:** ^1^ Institute for Transfusion Medicine and Immunohematology, Johann-Wolfgang-Goethe University and German Red Cross Blood Service, Frankfurt am Main, Germany; ^2^ Georg-Speyer-Haus, Institute for Tumorbiology and experimental Therapy, Frankfurt, Germany; ^3^ Department of Internal Medicine V, Hematology, Oncology and Rheumatology, University of Heidelberg, Heidelberg, Germany

**Keywords:** gene regulation, transcription factors, epigenetics, hematopoiesis, oncogenes

## Abstract

Hematopoietic differentiation is controlled by key transcription factors, which regulate stem cell functions and differentiation. TAL1 is a central transcription factor for hematopoietic stem cell development in the embryo and for gene regulation during erythroid/megakaryocytic differentiation. Knowledge of the target genes controlled by a given transcription factor is important to understand its contribution to normal development and disease. To uncover direct target genes of TAL1 we used high affinity streptavidin/biotin-based chromatin precipitation (Strep-CP) followed by Strep-CP on ChIP analysis using ChIP promoter arrays. We identified 451 TAL1 target genes in K562 cells. Furthermore, we analysed the regulation of one of these genes, the catalytic subunit beta of protein kinase A (*PRKACB*), during megakaryopoiesis of K562 and primary human CD34+ stem cell/progenitor cells. We found that TAL1 together with hematopoietic transcription factors RUNX1 and GATA1 binds to the promoter of the isoform 3 of *PRKACB* (*Cβ3*). During megakaryocytic differentiation a coactivator complex on the *Cβ3* promoter, which includes WDR5 and p300, is replaced with a corepressor complex. In this manner, activating chromatin modifications are removed and expression of the *PRKACB-Cβ3* isoform during megakaryocytic differentiation is reduced. Our data uncover a role of the TAL1 complex in controlling differential isoform expression of *PRKACB*. These results reveal a novel function of TAL1, RUNX1 and GATA1 in the transcriptional control of protein kinase A activity, with implications for cellular signalling control during differentiation and disease.

## INTRODUCTION

During hematopoiesis mature blood cells are generated from hematopoietic stem cells (HSC), which reside in the bone marrow. HSCs produce progeny of intermediate repopulation potential, the progenitor cells. Meanwhile, maintaining a finite stable pool of stem cells by self-renewal. Hematopoietic progenitor cells undergo further differentiation into mature cells of the lymphoid and myeloid branches. This differentiation process is influenced by internal and external signalling events and by the activity of transcription factors at the endpoint of these signalling pathways [1]. Transcription factors execute differentiation programs by activating cell type specific gene expression. The important function of transcription factors in hematopoiesis is reflected by the fact that loss of function of specific transcription factors can obliterate stem cell function and/or lead to altered lineage differentiation [2]. In humans, mutations and chromosomal translocations of transcription factors have been identified as a cause of leukemia and lymphoma [3]. Thus, extensive research in the field is aimed to understand the function of hematopoietic transcription factors in health and disease.

TAL1 (T-cell acute lymphocytic leukemia 1) is one of the central transcription factors for HSC development in the embryo and for gene regulation during erythroid/megakaryocytic differentiation [4–8]. Furthermore, aberrant expression of TAL1 in the T-cell compartment caused by chromosomal translocations leads to T-lineage acute lymphoblastic leukemia (T-ALL) [9–11]. Considerable progress has been made to better understand the function of TAL1 by the identification of TAL1 target genes [12–14]. Recently, we discovered that TAL1 interacts with RUNX1 (RUNT-related transcription factor 1) on erythroid genes in progenitor cells [15–17]. However, although TAL1 is readily expressed in erythrocyte/megakaryocyte progenitors and in the megakaryocytic lineage [18], little is known about the function of TAL1 in megakaryocytes.

To examine the role of TAL1 during megakaryopoiesis, we performed chromatin precipitation and promoter ChIP array analysis to define megakaryocytic target genes of TAL1. We identified a number of TAL1 target genes, which had recently been associated with megakaryocytic differentiation of K562 cells [19]. In particular, we found that TAL1 is a regulator of the catalytic subunit beta of protein kinase A (PRKACB). PKA is a cytoplasmatic holoenzyme consistent of two catalytic and two regulatory subunits. Upon generation of cyclic AMP (cAMP) e.g. upon hormone stimulus, cAMP binds to the regulatory units of PKA [20, 21]. This triggers dissociation of the regulatory and catalytic subunits. The catalytic subunits (Cα, Cβ, Cγ) phosphorylate substrates on serine and threonine residues [22]. Substrates include transcription factors, which have a role in differentiation and proliferation [20, 23]. The *PRKACB* gene encodes the beta subunit of PKA. There are four isoforms of *PRKACB* described and in addition several splice variants, each of the isoforms has a separate promoter and a distinct N-terminus [24, 25].

We found that TAL1 binds to the promoter of isoform 3 of *PRKACB* (*Cβ3*) together with the hematopoietic transcription factors RUNX1 and GATA1 (GATA-binding protein 1). Our data show that GATA1 acts as an activator of *Cβ3* isoform expression. Furthermore, RUNX1 and TAL1 contribute to the repression of *Cβ3* expression. Repression of *Cβ3* during megakaryocytic differentiation is triggered by the loss of coactivator binding and by recruitment of corepressor proteins to the *Cβ3* promoter.

## RESULTS

### Identification of TAL1 target genes

**Figure 1:**
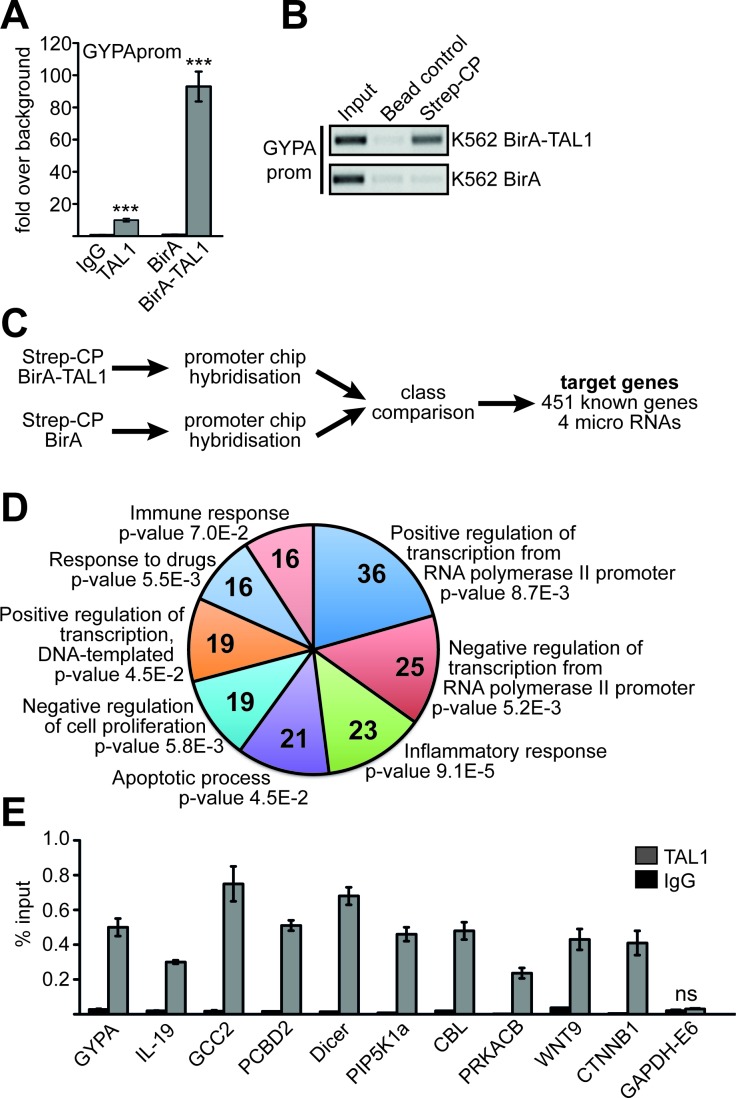
Identification of TAL1 target genes **(A)** Comparison of a chromatin immunoprecipitation (ChIP) using a TAL1 specific antibody and streptavidin/biotin chromatin precipitation (Strep-CP). ChIP-PCR is performed with primers specific for the promoter of the known TAL1 target *GYPA*. Values are given as fold over IgG background or as fold over background using a BirA-ligase only expressing K562 cell line (BirA) as control, respectively. Error bars represent the standard deviation from three independent evaluations. The P values were calculated using Student's t-test. ***P < .001. **(B)** Control Strep-CP from K562 cells harboring the BirA-ligase and BirA-TAL1 (upper part) compared to cells with BirA-ligase only (lower part). A precipitation with magnetic IgG beads was included as a negative control. ChIP-PCR was performed against *GYPA* promoter (GYPAprom). The PCR product was analysed on an agarose gel. The color inverted image of the gel is shown. **(C)** Flow chart showing the identification of TAL1 target genes by Strep-CP and ChIP analysis. **(D)** GO-term analysis using DAVID (DAVID Bioinformatics Resources 6.8) [33, 34] of the 451 potential TAL1 target genes. Shown are the eight GO-terms with the highest number of genes and the P values are given. Parameters for GO-term biological process (GO-BP direct) were default settings (with modified Fisher exact P value, EASE, set as 0.1). **(E)** Verification of TAL1 target genes found by Strep-CP using a standard ChIP with anti-TAL1 antibody. *GYPA* promoter was used as positive control and exon 6 of *GAPDH* (GAPDH-E6) as negative control. ChIP-PCR was performed with specific primer pairs against the given loci. Values are given as percent enrichment compared to the input. Error bars represent the standard deviation of two independent evaluations performed in duplicate. The P values for all verification ChIP experiments were at least P < .05 according to Student's t-test.

To identify TAL1 target genes, we made use of K562 cells. These cells exhibit properties of erythrocyte/megakaryocyte progenitor cells as they can be induced towards the erythroid or megakaryocytic lineage [26–28]. We established a BirA-TAL1 K562 cell line expressing TAL1 fused to a 12 amino acid BirA-tag, which is biotinylated within the cells by a coexpressed BirA-ligase [14]. With this system biotinylated BirA-tagged TAL1 protein can be purified with streptavidin coated beads [16, 29]. Alternatively, the BirA-tag/streptavidin interaction can be used for chromatin immunoprecipitation (ChIP), which has the advantage of high affinity without use of an antibody [14, 30, 31]. By streptavidin based chromatin precipitation (Strep-CP) we could enrich promoter DNA of the well-established TAL1 target gene *glycophorin A* (*GYPA*) 10-fold better than with a TAL1 antibody, despite more stringent washing conditions with Strep-CP (Figure 1A, 1B).

To identify novel TAL1 target genes, we performed ‘Strep-CP on ChIP’ analysis using ChIP-arrays with 12.000 human promoter regions spotted as oligonucleotides (Figure 1C) [32]. 451 promoter sites with TAL1 binding were identified at genes (Supplementary File 1). When we applied GO-term analysis using DAVID [33, 34] we found that genes involved in biological functions related to transcriptional regulation, inflammatory responses, apoptotic processes, negative regulation of cell proliferation, response to drugs and immune response were enriched in our dataset (Figure 1D, GO term IDs are given in Supplementary Figure 1). For further analysis we chose nine genes, which have a role in human disease. These genes also displayed changes in gene expression upon induction of megakaryocytic differentiation of K562 cells [19]. To verify TAL1 target genes, TAL1 binding was tested in wild type K562 cells with a standard ChIP using a TAL1 specific antibody and primers specific for the identified regions (Figure 1E). ChIP against the established TAL1 target *GYPA* served as positive control and ChIP against exon 6 of *GAPDH* as negative control. We confirmed TAL1 binding to regulatory elements of *IL-19* (interleukin 19), *GCC2* (GRIP and coiled-coil domain containing 2), *PCBD2* (pterin-4 alpha-carbinolamine dehydratase 2), *Dicer*, *PIP5K1a* (phosphatidylinositol-4-phosphate 5-kinase, type I, alpha), *CBL* (casitas B-lineage lymphoma), *PRKACB* (protein kinase, cAMP-dependent, catalytic, beta), *WNT9* (wingless-type MMTV integration site family, member 9) and *CTNNB1* (catenin (cadherin-associated protein) beta 1) (Figure 1E).

### Analysis of TAL1 target gene expression

**Figure 2:**
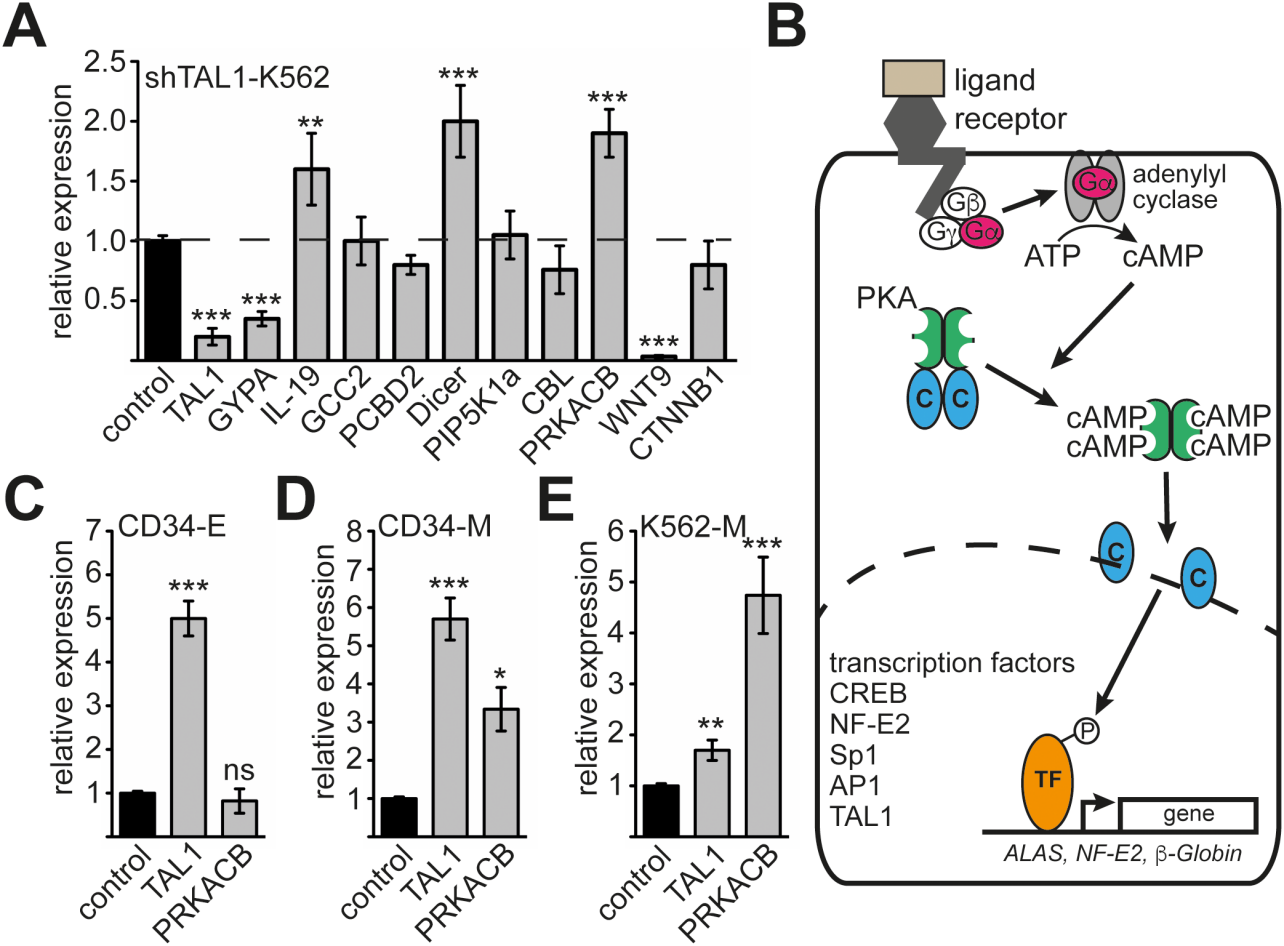
Analysis of *PRKACB* isoform expression during differentiation **(A)** Expression of nine selected TAL1 target genes upon TAL1 knockdown. TAL1 knockdown was performed by shRNA in K562 cells and the expression of the given genes measured by qRT-PCR. The well-established TAL1 target gene *GYPA* was included as a positive control. Data are shown as relative expression compared to values gathered upon transduction of a non-targeting shRNA vector (labelled as control). Expression of GAPDH served as internal control for normalization of gene expression. **(B)** Schematic presentation of protein kinase A (PKA) function. Upon activation of G-protein receptor the Gα subunit activates an adenylyl cyclase, which produces cAMP. Binding of cAMP to the regulatory units of PKA sets free the catalytic subunits, which translocate into the nucleus. In the nucleus several transcription factors are phosphorylated. Examples are CREB, NF-E2, Sp1, AP1 and TAL1. Some of these transcription factors are able to regulate erythroid genes, such as *ALAS*, *NF-E2* and *β-globin*. **(C)** Expression of *TAL1* and *PRKACB* during erythroid differentiation of primary human CD34+ progenitor cells. **(D)** Expression of *TAL1* and *PRKACB* during megakaryocytic differentiation of primary human CD34+ progenitor cells. **(E)** Expression of *TAL1* and *PRKACB* during megakaryocytic differentiation of K562 cells. **(C-E)** Values are shown as relative expression compared to undifferentiated cells labelled as control. Expression of GAPDH served as internal control for normalization of gene expression. Error bars represent the standard deviation of qRT-PCR evaluations of three independent experiments measured in duplicates. The P values were calculated using Student's t-test. **P < .01; ***P < .001.

To evaluate which of the genes bound by TAL1 respond to changes in TAL1 expression, we performed knockdown of TAL1 by shRNA in K562 cells. We found that *IL-19*, *Dicer* and *PRKACB* were upregulated, whereas *WNT9* was downregulated upon Tal1 knockdown. The other genes did not exhibit expression changes upon TAL1 knockdown under these conditions (Figure 2A). This observation is in line with the notion that TAL1 can serve as an activator or repressor of gene expression in a cell- and promoter-dependent context. PRKACB is an enzymatic subunit of the protein kinase A complex (PKA). PKA is a very important signalling molecule, which influences almost all aspects of cellular differentiation through phosphorylation of transcription factors (Figure 2B).

PRKACB is involved in PKA-signalling, which is a central signalling pathway within cells. Furthermore, PKA-signalling influences the function of TAL1 [35, 36]. Thus, the potential involvement of TAL1 in regulating PRKACB opens the possibility of a reciprocal regulation between PKA-signalling and the functional regulation of TAL1. For this reason we further studied the influence of TAL1 on *PRKACB* expression. To further investigate *PRKACB* expression at the erythroid/megakaryocytic branching point, we used primary human CD34+ progenitor cells. We differentiated these cells towards the erythroid or megakaryocytic lineage [14, 37]. Overall expression of *PRKACB* was not altered during erythroid differentiation (Figure 2C) and increased during megakaryocytic differentiation (Figure 2D). Similarly, megakaryocytic differentiation of K562 cells led to an increased *PRKACB* mRNA (Figure 2E) [19].

### Isoform 3 of *PRKACB* is down regulated during megakaryocytic differentiation

**Figure 3:**
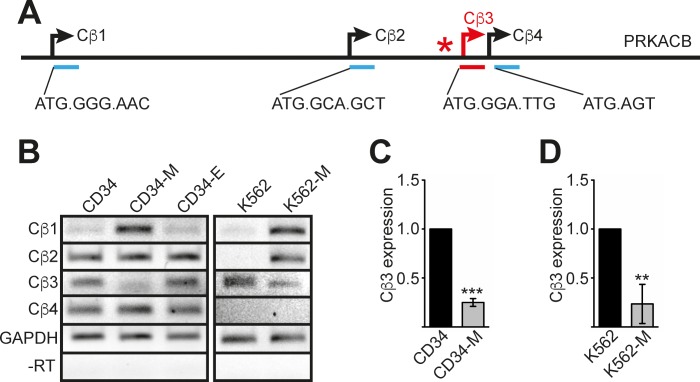
*PRKACB* isoform expression during differentiation **(A)** Schematic overview of the *PRKACB* locus. The four promoter regions of the different isoforms are underlined. The first base pairs and the amino acid sequences of the four *PRKACB* isoforms (*Cβ1-Cβ4*) are given. **(B)** Semiquantitative RT-PCR of the different *PRKACB* isoforms during CD34+ megakaryocytic (CD34-M) or erythroid (CD34-E) differentiation and TPA-induced megakaryocytic differentiation of K562 cells (K562-M). Expression of GAPDH served as internal control and a –RT control for GAPDH is shown. **(C)** Quantitative RT-PCR evaluation of Cβ3 expression during megakaryocytic differentiation of human CD34+ cells (CD34-M). **(D)** Quantitative RT-PCR evaluation of *Cβ3* expression during megakaryocytic differentiation of K562 cells (K562-M). Error bars represent the standard deviation of qPCR evaluations of three independent experiments measured in duplicates. P values were calculated using Student's t-test. ***P < .001.

The *PRKACB* gene encodes the catalytic beta subunit of PKA. Four isoforms of *PRKACB* have been described in addition to several splice variants [24]; each of the isoforms has a separate promoter and a distinct N-terminus (Figure 3A). Based on the ‘Strep-CP on ChIP’ analysis data, the TAL1 binding site is located 5′ of the *PRKACB* isoform 3 (*Cβ3*) (Figure 3A). To analyse the role of TAL1 on Cβ3 expression control, we examined expression of the different isoforms using isoform specific PCR primer and semiquantitative RT-PCR. Interestingly, upon megakaryocytic differentiation of hCD34+ or K562 cells expression of the *PRKACB* isoform 1 (*Cβ1*) was increased, whereas the *Cβ3* isoform was decreased (Figure 3B). The decreased expression of the *Cβ3* isoform upon megakaryocytic differentiation was verified in hCD34+ and K562 cells using qRT-PCR (Figure 3C-3D).

### Analysis of the *PRKACB*-β3 promoter

**Figure 4:**
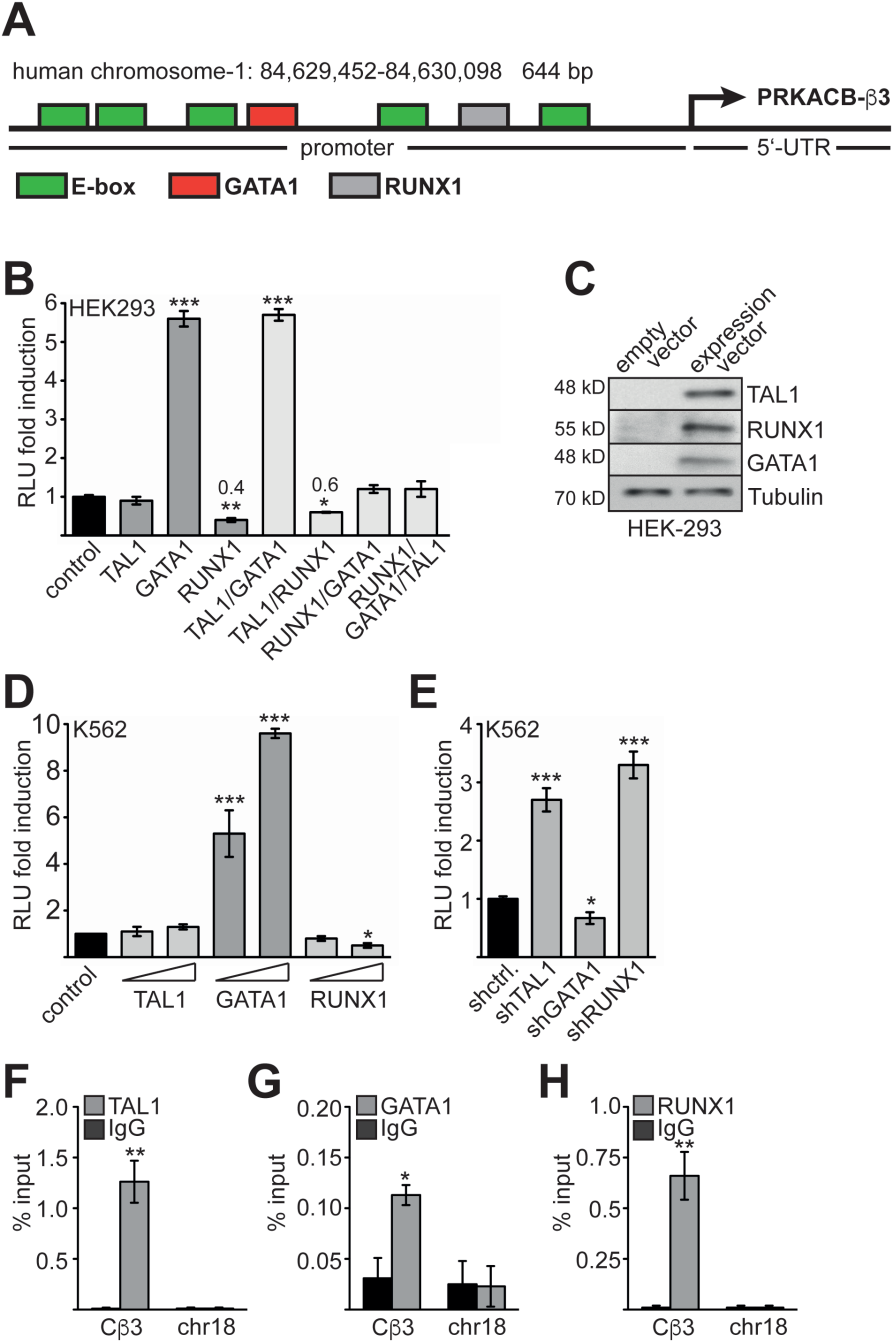
Analysis of the Cβ3 promoter **(A)** Schematic representation of the promoter and 5′-UTR of the human *PRKACB*- *Cβ3* isoform. The region was cloned in a luciferase vector. Identified potential binding sites for hematopoietic transcription factors and the mRNA start are labelled. **(B)** Luciferase promoter assay of the *Cβ3* promoter in HEK293 cells. Cotransfection of the *Cβ3* promoter with transcription factors TAL1, RUNX1 and GATA1. **(C)** The integrity of the overexpression constructs was verified by western blot. **(D)** Luciferase promoter assay of the *Cβ3* promoter in K562 cells upon overexpression of TAL1, GATA1 and RUNX1, respectively. Raising amounts of expression vectors were transfected. **(E)** Luciferase promoter assay of the *Cβ3* promoter in K562 cells upon knockdown of TAL1, GATA1 and RUNX1, respectively. Luciferase activity was measured two days after transfection. Relative light units (RLU) are given as fold induction compared to the activity of the promoter only (control). **(F)** ChIP assay showing the binding of TAL1 to the *Cβ3* promoter in K562 cells. ChIP-PCR with primers against an unrelated region on chromosome-18 (chr18) serves as negative control. **(G)** ChIP assay showing the binding of GATA1 to the *Cβ3* promoter in K562 cells. ChIP-PCR with primers against an unrelated region on chromosome-18 (chr18) serves as negative control. **(H)** ChIP assay showing the binding of RUNX1 to the *Cβ3* promoter in K562 cells. ChIP-PCR with primers against an unrelated region on chromosome 18 (chr18) serves as negative control. Error bars represent the standard deviation of at least three determinations. The P values were calculated using Student's t-test. *P < .05; **P < .01; ***P < .001.

Using TESS (transcription element search system) software [38] we analysed the 5′ region of the *Cβ3* isoform and identified several potential binding sites for TAL1 (E-Boxes) 5′ of the transcription start. Furthermore, RUNX1 and GATA1 sites were present in this area (Figure 4A). We cloned the 5′-region including the 5′-UTR of *Cβ3* into the pGL4 luciferase reporter gene vector for promoter analysis. Subsequently, we cotransfected the promoter reporter construct with TAL1, GATA1 or RUNX1 into HEK293 cells. Cotransfection of TAL1 with the promoter construct did not influence promoter activity, whereas GATA1 markedly activated the promoter (Figure 4B). Interestingly, RUNX1 repressed *Cβ3* promoter activity. Cotransfection experiments revealed that RUNX1 inhibits the activity of the *Cβ3* promoter even in the presence of GATA1 or TAL1 (Figure 4B). The expression constructs were verified by western blot (Figure 4C). Subsequently, we performed a reporter gene assay in K562 cells and detected an activating effect of GATA1 on reporter gene activity and a repressive effect of RUNX1 (Figure 4D). Furthermore, cotransfection of knockdown constructs against TAL1 and RUNX1 increased *Cβ3* promoter activity. In contrast, GATA1 knockdown resulted in decreased activity (Figure 4E). These data indicate that GATA1 activates the *PRKACB* β3 promoter, whereas RUNX1 is a repressor of the *PRKACB* β3 isoform. Subsequently, we showed by ChIP that TAL1, GATA1 and RUNX1 are present at the *PRKACB* β3 isoform promotor but not to an unrelated region on chromosome-18 (chr18) (Figure 4F-4H).

### TAL1, GATA1 and RUNX1 influence endogenous *PRKACB* expression

**Figure 5:**
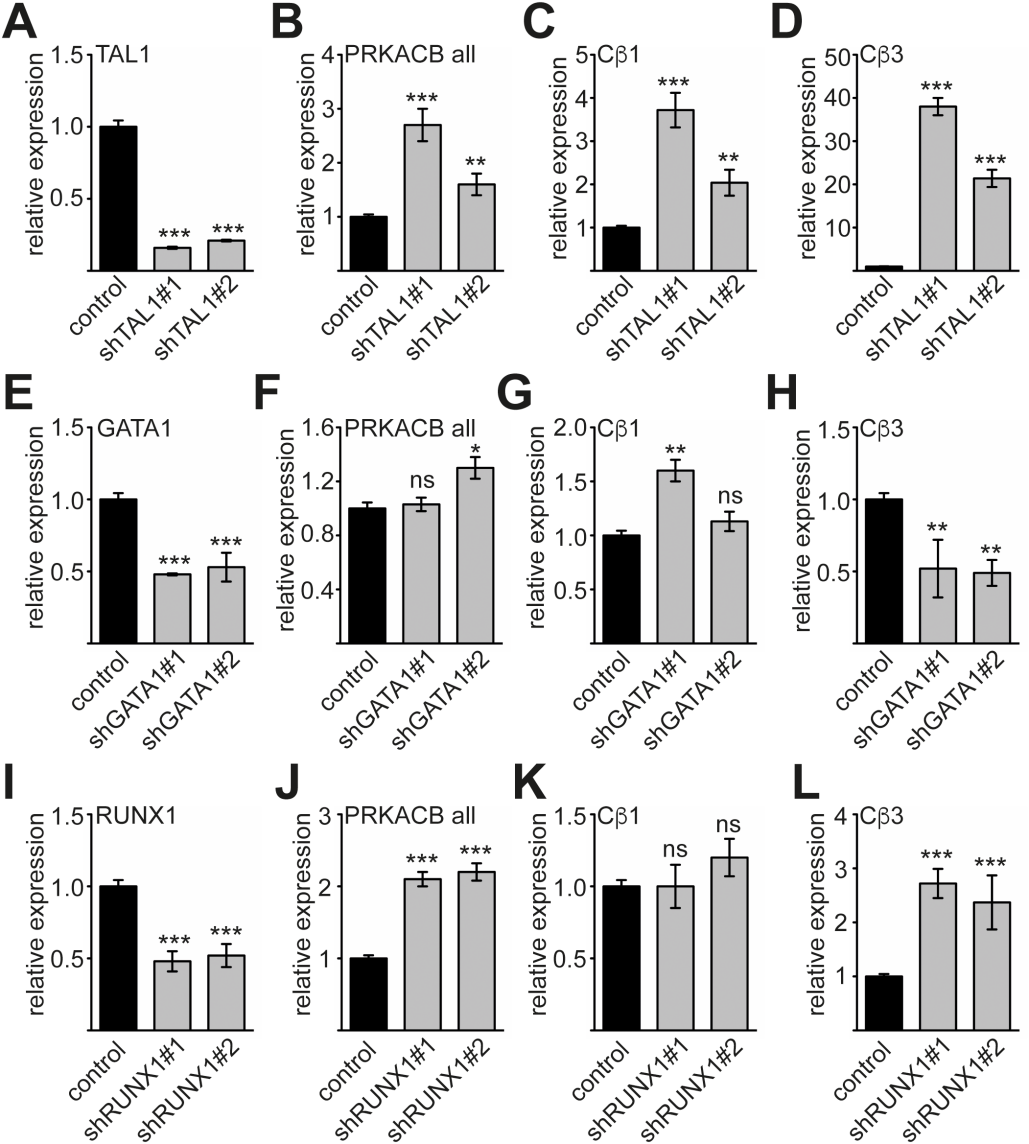
Expression of Cβ3 isoform is influenced by TAL1, GATA1 and RUNX1 **(A)** Knockdown of TAL1 by two different shRNAs measured by qRT-PCR. **(B)** Overall expression of *PRKACB* (PRKACB all) upon TAL1 knockdown. **(C)** Expression of *Cβ1* isoform upon TAL1 knockdown. **(D)** Expression of *Cβ3* isoform upon TAL1 knockdown. **(E)** Knockdown of GATA1 by two different shRNAs measured by qRT-PCR. **(F)** Overall expression of *PRKACB* upon GATA1 knockdown. **(G)** Expression of *Cβ1* isoform upon GATA1 knockdown. **(H)** Expression of *Cβ3* isoform upon GATA1 knockdown. **(I)** Knockdown of RUNX1 by two different shRNAs measured by qRT-PCR. **(J)** Overall expression of *PRKACB* upon RUNX1 knockdown. **(K)** Expression of *Cβ1* isoform upon RUNX1 knockdown. **(L)** Expression of Cβ3 isoform upon RUNX1 knockdown. Values are shown as relative expression compared to cells transduced with the corresponding vector expressing a non-targeting shRNA labelled as control. Expression of GAPDH served as internal control for normalization of gene expression. The error bars give the standard deviation of at least three evaluations performed in duplicates. The P values were calculated using Student's t-test. *P < .05; **P < .01; ***P < .001.

Because TAL1, GATA1 and RUNX1 play a major role during megakaryocytic differentiation [15] and our data hint towards a role of these transcription factors in regulation of PRKACB, we knocked down the transcription factors by shRNA (Figure 5). TAL1 knockdown (Figure 5A) resulted in increase of overall *PRKACB* expression (Figure 5B). The expression of the *Cβ1* isoform of *PRKACB* was modestly increased about 2- to 4-fold (Figure 5C), whereas expression of the *Cβ3* isoform increased substantially (Figure 5D). This result hints towards a repressive function of TAL1 on the expression of the *Cβ3* isoform. Given the variation gathered with the different GATA1 sh-constructs the knockdown of GATA1 did not alter overall *PRKACB* expression and did not significantly change *Cβ1* expression (Figure 5E-5G). However, *Cβ3* isoform expression was reduced upon GATA1 knockdown (Figure 5H). This is in line with our reporter gene assay showing that GATA1 is an activator of the *Cβ3* promoter (Figure 3B, 3D). The knockdown of RUNX1 (Figure 5I) resulted in an overall upregulation of *PRKACB* expression (Figure 5J). Interestingly, expression of *Cβ1* was unchanged upon RUNX1 knockdown (Figure 5K), whereas *Cβ3* expression was increased (Figure 5L). Taken together, we detected differential effects of TAL1, GATA1 and RUNX1 on *PRKACB* isoform expression. Of note, *Cβ3* expression was increased by knockdown of TAL1 and RUNX1 indicating a repressive role of these transcription factors. By contrast, GATA1 knockdown reduced *Cβ3* expression indicating an activating role of GATA1 on the expression of this isoform.

### Cofactor exchange on the *PRKACB* promoter upon differentiation

**Figure 6:**
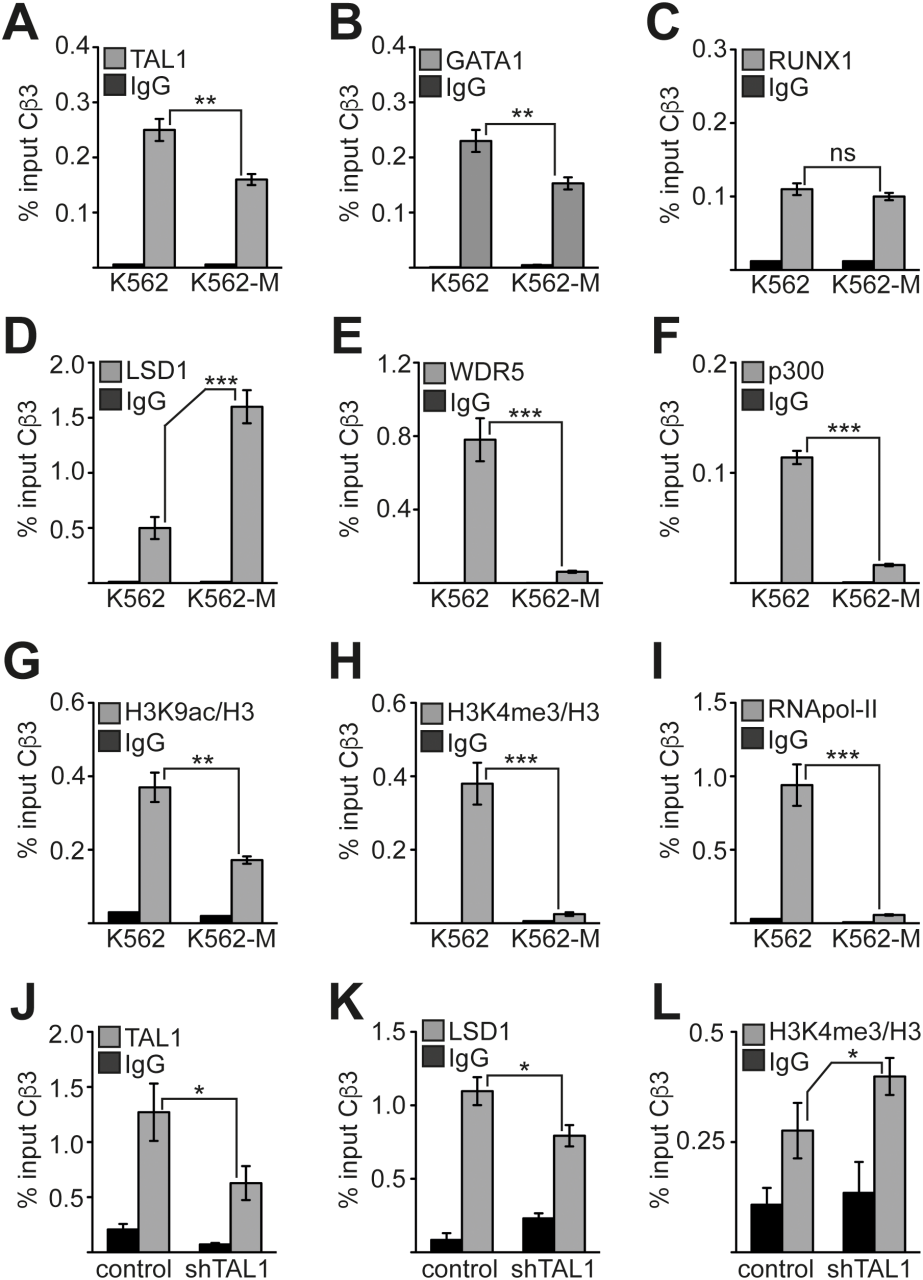
Changes at the Cβ3 promoter during megakaryocytic differentiation **(A-C)** Binding of transcription factors TAL1, GATA1 and RUNX1 to the promoter of Cβ3 isoform before and after megakaryocytic differentiation of K562 cells induced by TPA. **(D-F)** Binding of the chromatin modifying cofactors LSD1, WDR5 and p300 to the promoter of *Cβ3* isoform before and after megakaryocytic differentiation of K562 cells induced by TPA. **(G-H)** Measurement of the histone modification status at the *Cβ3* promoter. The H3K9ac and H3K4me3 status was determined before and after megakaryocytic differentiation of K562 cells induced by TPA. The values were corrected to the histone density according to a ChIP against histone 3 (H3). **(I)** Binding of RNA-polymerase II (RNApol-II) to the *Cβ3* promoter. ChIP was performed with specific antibodies in untreated and TPA-treated (96h) K562 cells. **(J)** ChIP assay upon TAL1 knockdown. TAL1 binding to the *Cβ3* promoter was reduced upon TAL1 knockdown in K562 cells. **(K)** LSD1 binding to the *Cβ3* promoter was reduced upon TAL1 knockdown in K562 cells. **(L)** H3K4me3 at the *Cβ3* promoter was increased upon TAL1 knockdown in K562 cells. Values are given as percent enrichment compared to the input. Error bars represent the standard deviation of three determinations measured in duplicates. The P values were calculated using Student's t-test. **P < .01; ***P < .001.

Transcriptional expression of the *Cβ3* isoform of *PRKACB* is downregulated during megakaryocytic differentiation. To determine the molecular mechanism underlying this downregulation and the individual contributions of TAL1, GATA1 and RUNX1, we performed ChIP analysis before and after megakaryocytic differentiation of K562 cells. We examined transcription factor binding to the *Cβ3* promoter and could detect TAL1 on the *Cβ3* promoter before and, less strongly, after megakaryocytic differentiation (Figure 6A). Similarly, GATA1 occupancy was reduced upon megakaryocytic induction (Figure 6B) whereas RUNX1 occupancy was unaffected (Figure 6C). When we induced K562 cells towards megakaryocytic differentiation, we observed an increase of LSD1 occupancy on the *Cβ3* promoter (Figure 6D). LSD1 is able to remove methyl residues from lysine 4 of histone 3, and thus counteracts H3K4 methylation by the oncogenic MLL complex. Thus, LSD1 can act as a repressor by decreasing the activating H3K4me3 mark on a locus. Concomitantly, binding of WDR5 was strongly reduced (Figure 6E). WDR5 is a central component of the WDR5/MLL complex, which mediates H3K4me3. In parallel, we found reduced levels of the histone acetyltransferase p300 on the promoter (Figure 6F). Consistent with the changes in histone modifying cofactor occupancy we detected a decrease of the activating histone modifications H3K9ac and H3K4me3 at the *Cβ3* promoter (Figure 6G-6H) and a concomitant decrease of RNA polymerase II (RNApol-II) occupancy (Figure 6I). To determine if TAL1 contributes to the recruitment of LSD1 to the *Cβ3* promoter, we performed a ChIP-Assay upon TAL1 knockdown in K562 cells. The TAL1 knockdown resulted in less TAL1 occupancy (Figure 6J) and contributed to reduced presence of LSD1 (Figure 6K), concomitantly H3K4me3 was increased (Figure 6L). This suggests that TAL1 at least partly contributed to LSD1 recruitment in K562 cells. Upon megakaryocytic differentiation the downregulation of *Cβ3* expression is triggered by an exchange of an activating cofactor complex containing CBP/p300 and WDR5 with a repressive complex containing increased LSD1 levels.

## DISCUSSION

The transcription factor TAL1 is an important regulator of gene expression at the erythroid/megakaryocytic branching. To better understand the function of TAL1 at this important differentiation point, we identified target genes of TAL1 using a biotin-tagged TAL1 in K562 cells. For more detailed analysis we focused on the *PRKACB* gene, which encodes the catalytic beta subunit of the protein kinase A (PKA). PKA-signalling is important at the megakaryocytic/erythroid branching [39]. Furthermore, it influences downstream transcription factors such as TAL1 [35, 36]. In particular, we could demonstrate that isoform 3 of *PRKACB* (*Cβ3*) is a direct target gene of the hematopoietic transcription factors TAL1, RUNX1 and GATA1. Interestingly, expression of *PRKACB Cβ3* is upregulated during erythroid differentiation and downregulated during megakaryopoiesis. In this respect, *Cβ3* behaves like an erythroid gene. Recently, we reported that the transcription factor RUNX1 is a suppressor of erythroid TAL1 target genes such as *KLF1* and the microRNA miR-144/451 [15, 17]. Similar to this observation, we found that RUNX1 acts as repressor of the *PRKACB Cβ3* isoform. On the other hand, our data suggest an activating role of the transcription factor GATA1 on *Cβ3* isoform expression.

We showed that TAL1, GATA1 and RUNX1 were all already present at the promoter before megakaryocytic specification and remained there during and after differentiation. However, TAL1 and GATA1 binding was decreased upon megakaryocytic differentiation. It had been described that TAL1 and GATA1 can be found together on GATA1/E-box composite elements, which assembles a multiprotein complex with GATA1, LDB1, LMO2, TAL1 and E2A in erythroid cells [40–43]. Similarly, GATA1 and RUNX1 have been shown to cooperate during megakaryocytic differentiation on the *αII integrin* promoter [26]. Furthermore, we recently found that RUNX1 can be a repressor or activator of megakaryocytic genes depending of the differentiation status [37]. Interestingly, RUNX1 and TAL1 are able to directly interact on the erythroid gene *KLF1*, and during megakaryocytic differentiation epigenetic corepressors are recruited dependent on the presence of RUNX1 [15].

Another observation of ours is that megakaryocytic differentiation is accompanied by a cofactor exchange at the *Cβ3* promoter. Decreased *Cβ3* expression correlates with reduced presence of the coactivator proteins WDR5 and p300 (Figure 6E–6F). Concomitantly, occupancy of the *Cβ3* promoter by the histone lysine demethylase LSD1 increases (Figure 6D). In line with this observation the histone modification pattern changes from an activating state with high H3K9ac and H3K4me3 to a repressive one with lower H3K9ac and loss of H3K4me3 (Figure 6G, 6H). TAL1 was shown to be able to directly recruit LSD1 [44] and thus, might be involved in the recruitment of LSD1 on the *Cβ3* promoter. Indeed, knockdown of TAL1 in undifferentiated K562 cells led to decreased LSD1 occupancy and increased H3K4me3 (Figure 6J-6L), showing that TAL1 contributes to repression of *Cβ3*. Most interestingly, PKA-signalling itself can abolish the association of TAL1 with LSD1 by phosphorylation of serine 172 [36]. This modification destabilizes TAL1-LSD1 interaction and may lead to altered TAL1 DNA binding activity [35]. It is conceivable that TAL1 itself is phosphorylated by cAMP dependent phosphorylation at serine 172 during megakaryopoiesis leading to increased LSD1 recruitment. At present no ChIP-grade TAL1Sp172 specific antibody is available to test this idea at the endogenous protein level. However, other transcription factors, such as RUNX1 or GATA2, are also able to interact with LSD1 and might contribute to LSD1 recruitment [45–47].

Furthermore, downstream of PKA signalling are important transcription factors like cAMP-response element-binding protein (CREB) [48], nuclear factor kappa light chain enhancer of activated B-cells (NF-κB) [49, 50], the erythroid nuclear factor 2 (NF-E2) [51], the specificity protein (Sp1) [52] and the activator protein 1 (AP1) [53]. By influencing *PRKACB* isoform expression, TAL1, RUNX1 and GATA1 could impact on cAMP signal transduction pathways. This is interesting because cAMP signalling can induce both proliferative and anti-proliferative effects, depending on the cell type [54, 55]. It is plausible that the relative concentrations of the different isoforms of *PRKACB* are a determinant of the context-dependent outcome of cAMP signalling on proliferation. Since TAL1 is associated with increased cell proliferation during erythroid differentiation, it is possible that TAL1 may exert part of its proliferative effect by influencing signalling. Moreover, it was shown that cAMP signalling is implicated in the repression of megakaryocytic differentiation [39]. However, at this time point it is unclear how the distinct PRKACB isoforms influence differentiation. It is conceivable that the altered N-terminus of the different PRKACB isoforms could impact substrate specificities resulting in altered biological functions [25, 56, 57].

It was also discussed that cAMP signalling plays a role in malignant hematopoiesis [58]. Therefore, our finding that hematopoietic transcription factors, such as TAL1 and RUNX1, influence expression of *PRKACB* isoforms could connect aberrant transcription factor function with altered PKA signalling in leukemia. This would be supported by the observation that *PRKACB* regulation by the important oncogene c-Myc is involved in the control of proliferation [59]. Our observations open the possibility of a regulatory loop between transcriptional regulation of *PRKACB* isoform expression and the modification status of TAL1 in differentiation and leukemia.

## MATERIALS AND METHODS

### Cell culture

K562 cells (ATCC) were maintained in RPMI with 10% FCS and 1% penicillin/streptomycin. To induce megakaryocytic differentiation, K562 cells were treated with 30 nM phorbol 12-o-tetradecanoylphorbol-13-acetate (TPA). BirA-K562 cells were established as described [14]. HEK293 cells (ATCC) were cultured in DMEM with 10% FCS and 1% penicillin/streptomycin. Mobilized peripheral human primary CD34+ cells were obtained with written informed consent from healthy donors from the Institute for Transfusion Medicine Frankfurt following the ethical guidelines of the Frankfurt University (permit #329-10 of the Frankfurt University Clinic Ethikkommission). The cells were expanded *in vitro* under serum-free conditions and subjected to erythroid or megakaryocytic differentiation. Expansion took place for 6 days in StemSpan medium (Stem cell Technologies) supplemented with Flt-3 ligand (100 ng/ml), SCF (100 ng/ml), IL-3 (20 ng/ml) and IL-6 (20 ng/ml). For erythroid differentiation cells were transferred into StemSpan with SCF (20 ng/ml), IL-3 (5 ng/ml), dexamethasone (2 mM, Sigma), estradiol (0.2 mM, Sigma) and erythropoietin (1 unit/ml, Applichem). For megakaryocytic differentiation cells were treated with SCF (1 ng/ml), TPO (30 ng/ml), IL-9 (13.5 ng/ml), and IL-6 (7.5 ng/ml) for 4-6 days [60].

### Knockdown experiments with shRNA

Knockdown of TAL1, RUNX1 and GATA1 was achieved with shRNAs present in the pGIPZ vector (Open Biosystems) [17]. The accession numbers of the pGIPZ constructs is given in Supplementary Material. Controls were expressing non-targeting shRNA from the corresponding backbone. Knockdown was verified by qRT-PCR (Figure 5). A corresponding western blot from the same lysates showing the decreased protein amount of TAL1, RUNX1 and GATA1 is shown in [17]. For western blot analysis whole cell extracts from transfected HEK293 cells were used. Proteins were separated using SDS-PAGE and transferred to PVDF membranes. Membranes were blocked with Roti-Block (Carl Roth) and exposed to primary antibody dilution over night at 4°C. Afterwards membranes were incubated with appropriate secondary antibodies coupled to horseradish peroxidase (HRP) for 1h at room temperature. Signals were visualized using Western Super ECL reagent (Pierce) and analysed using X-ray film. Immunoblotting analysis was performed using anti-TAL1 (sc-12984, Santa Cruz), anti-RUNX1 (ab23980, Abcam), anti-GATA1 (sc-1233, Santa Cruz) and anti-Tubulin (ab7291, Abcam).

### Reporter gene assay

The 5′-region of the *Cβ3* isoform was amplified from genomic DNA using phusion polymerase (New England Biolabs) and inserted in the luciferase vector pGL4.10 (Promega). Luciferase activity was measured 48h after transfection and normalised to the activity of a cotransfected CMV-promoter driven β-galactosidase vector. Data represent three independent experiments, performed in duplicates. Error bars show the standard deviation. Transfections were performed with metafectene according to the manufacturer's instructions.

### ChIP assay

1×10^7^ K562 cells were harvested and resuspended in 10 ml RPMI medium and cross-linked for 15 min with 0.75% formaldehyde. The cross-link was stopped by adding glycine to a final concentration of 0.125 M, the cells were washed with phosphate-buffered saline and lysed in 1.2 ml lysis buffer for 30 min on ice (50 mM HEPES-KOH, pH 7.5, 140 mM NaCl, 1 mM EDTA, pH 8.0, 1% Triton X-100, 0.1% Sodium Deoxycholate, 1% SDS, protease inhibitors). Chromatin was sheered using a bioruptor sonification device (Diagenode) resulting in fragments with an average of 500 bp length. Subsequently, 450 μl RIPA buffer (50 mM Tris, pH 8.0, 150 mM NaCl, 2 mM EDTA, pH 8.0, 1% NP-40, 0.5% sodium deoxycholate, protease-inhibitors), 5 μl blocked protein-G magnetic beads (Dynabeads Protein G-100, Invitrogen) and 2-10 μg antibody (or IgG as control) were added to 150 μl of chromatin and incubated over night at 4°C on a rotating wheel. One sample was spared for input control. The beads were captured using a magnetic rack and washed three times with wash buffer (20 mM Tris-HCl, pH 8.0, 150 mM NaCl, 0.1% SDS, 1% Triton X-100, 2 mM EDTA, pH 8.0) for 10 min on a rotating wheel at 4°C and once with final wash buffer (20 mM Tris-HCl, pH 8.0, 500 mM NaCl, 0.1% SDS, 1% Triton X-100, 2 mM EDTA, pH 8.0) for 15 min. DNA was eluted using elution buffer (1% SDS, 100 mM NaHCO_3_) at room temperature for 15 min with shaking. The cross-link was reversed in 400 μl elution buffer with 5 μl proteinase K (20 mg/ml, Applichem) for 5-6 hours at 65°C. The DNA was extracted using phenol-chloroform and precipitated over night at −20°C and washed with 70% ethanol. Antibodies used: anti-RUNX1 from Abcam (ab23980), anti-TAL1 from Abcam (ab75739), anti-PRMT6 from Santa Cruz (sc-55702), anti-p300/CBP from NeoMarkers (MS-586), anti-LSD1 from Abcam (ab17721), anti-WDR5 from Santa Cruz (sc-100895), anti-GATA1 from Santa Cruz (sc-1233) and anti-RNApol IIa from Abcam (ab5408). For the ChIP-Assay shown in Figure 4F-4H and Figure 6J-6K the following alternative antibodies were used: anti-GATA1 from Abcam (ab11963), anti-TAL1 from OriGene (GAT, TA590662-OR), anti-RUNX1 from Abcam (ab23980, batch GR275602-1). For chromatin immunoprecipitation of histone modifications antibodies from Abcam were used: anti-H3K9ac (ab10812) anti-H3K4me3 (ab1012) and anti-H3 (ab1791). Primer pairs used for ChIP-PCR are given in Supplementary Material.

### Strep-CP

Streptavidine/biotin-Chromatin-Precipitation (Strep-CP) was performed as described [14]. Following Strep-CP, we performed ‘Strep-CP on ChIP’ analysis using ChIP-arrays with 12.000 human promoter regions spotted as oligonucleotides [32]. For this, DNA from three independent Strep-CPs were linearly amplified and hybridized to the promoter array. Following intra-array and inter-array normalisation, TAL1 bound sequences were identified by class comparison of BirA-TAL1 cells with the BirA-ligase only control. 451 promoter sites with TAL1 binding were identified at genes with a high to low statistical likelihood of being target genes of TAL1 (Supplementary File 1). ‘Strep-CP on ChIP’ data are given. Raw data are given in Supplementary File 1. Genes were subjected to gene ontology (GO-term) analysis using DAVID (DAVID Bioinformatics Resources 6.8) [33, 34]. Parameters for GO-term biological process (GO-BP direct) were default settings (with modified Fisher exact P-value, EASE, set as 0.1).

### Quantitative PCR

Total mRNA was prepared using the RNeasy purification kit (Qiagen) and synthesis of cDNA was performed with omniscript reverse transcriptase (Qiagen). Specific primers were used for quantitative RT-PCR. PRKACB (PRKACB-forward: 5′-GCCACGACAGATTGGATTG-3′; PRKACB-reverse: 5′-TCCAGAGCCTCTAAAC TTTGGT-3′). For isoform specific PRKACB RT-PCR forward primer were designed from the isoform specific 5′-regions and the specific exons and one reverse primer was used for all isoforms (Cβ1-forward: 5′-ACTGTGGAGTGGCGGGCAC-3′; Cβ2-forward: 5′-TTGGAAGGTTTTGCTAGCCGGTT-3′; Cβ3-forward: 5′-TTGCCAGGTTCAACATGGGATT-3′, Cβ4-forward: 5′-GGAAAGGTTGGTTTTCATCATG-3′; Cβ1, 2, 3, 4-reverse: 5′-CTGAGTTGGATTCTCCCATTTT-3′). Primer pairs used for qRT-PCR are given in Supplementary Material. Quantitative PCR was performed using SYBR-green (Eurogentec) and a LightCycler 480 (Roche).

## SUPPLEMENTARY FIGURE AND FILES






