# Supplementary material for: Hematopoietic transcription factors and differential cofactor binding regulate PRKACB isoform expression
Source: Oncotarget. 2017 Apr 24;8(42):71685–98. doi: 10.18632/oncotarget.17386 (PMC5641081; doi:10.18632/oncotarget.17386)
Supplement: Supplementary file 1 [file oncotarget-08-71685-s001.pdf]

Hematopoietic transcription factors and differential cofactor binding regulate *PRKACB* isoform expression

SUPPLEMENTARY MATERIALS

GOTERM_BP_direct			
Term	Count	p-value	GO-ID
Positive regulation of transcription from RNA polymerase II promoter	36	8,7E-3	0045944
Negative regulation of transcription from RNA polymerase II promoter	25	5,2E-3	0000122
Inflammatory respone	23	9,1E-5	0006954
Apoptotic process	21	4,5E-2	0006915
Negative regulation of cell proliferation	19	5,8E-3	0008285
Positive regulation of transcription, DNA-templated	19	4,5E-2	0045893
Response to drugs	16	5,5E-3	0042493
Immune Response	16	7,0E-2	0006955

Supplementary Figure 1: GOTERM-IDs Given are the GO-IDs of the GO-terms shown in Figure 1D.

Supplementary Data File: Strep-CP on ChIP data

See Supplementary File 1

Supplementary Material:

See Supplementary File 2
