# Supplementary material for: Hematopoietic transcription factors and differential cofactor binding regulate PRKACB isoform expression
Source: Oncotarget. 2017 Apr 24;8(42):71685–98. doi: 10.18632/oncotarget.17386 (PMC5641081; doi:10.18632/oncotarget.17386)
Supplement: Supplementary file 3 [file oncotarget-08-71685-s003.docx]

**Supplementary Material Kuvardina et al**

**ChIP Primers**

hPRKACB

5’-CTGTTCACCATGCTACCCAGTC-3’

5’-ACAGTGTCAGATGCCATCCATGTG-3’

hGYPA

5’-CAGGCGCTTAACAACTTGCATCA-3’

5’-CATACATCCTGAGATCATGAGCTG-3’

hIL-19

5’-GGCTCTTCATTCATAGGTGC-3’

5’-GTCCACAACAGCACCACTTC-3’

hGCC2

5’-CACCATGCTGGCCACAAATG-3’

5’-CTCGGTTTCCACGTCTGTAA-3’

hPCBD2

5’-ATTACAGGCCGGGTTTCCA-3’

5’-CCTAAGTCTTGCAGTAGTGG-3’

hDicer

5’-CCACTGAAGAGATGTCTACATT-3’

5’-AGATGGTGGCCCTCTGTTTG-3’

hPIP5K1a

5’-GTAGGGACTGTCGGCCAAC-3’

5’-GCATCAGCCCACTCATTGG-3’

hCBL

5’-TATACTCGAGACCTGTGGTGCAATGCT-3’

5’-TATAAAGCTTGCGGACCGGCTTACTGG-3’

hWNT9

5’-ACACCAGCCATCGGGCAACG-3’

5’-GCTGCCTGGGTGTGCACCTT-3’

hCTNNB1

5’-GCCGAGTCCTGCAGCTGCTC-3’

5’-CGCTGTGGTCTGCATCGGGG-3’

hGAPDH-E6

5’-GCCAAGGCTGTGGGCAAGGT-3’

5’-CCTCCGACGCCTGCTTCACC-3’

**qRT-PCR Primers**

hRUNX1

5’-TCGACTCTCAACGGCACCCGA-3’

5’-TGACCGGCGTCGGGGAGTAG-3’

hTAL1

5’-TCGGCAGCGGGTTCTTTGGG-3’

5’-CCATCGCTCCCGGCTGTTGG-3’

hGATA1

5’-GACACTCCCCAGTCTTTCAGG-3’

5’-CAGTTGAGGCAGGGTAGAGC-3’

hGYPA

5’-CCCTCCAGAAGAGGAAACCGGAGA-3’

5’-GGCACGTCTGTGTCAGGTGAGG-3’

hPRKACB

5’-GCCACGACAGATTGGATTG-3’

5’-TCCAGAGCCTCTAAACTTTGGT-3’

Cβ1

5’-ACTGTGGAGTGGCGGGCAC-3’

5’-CTGAGTTGGATTCTCCCATTTT-3’

Cβ2

5’-TTGGAAGGTTTTGCTAGCCGGTT-3’

5’-CTGAGTTGGATTCTCCCATTTT-3’

Cβ3

5’-TTGCCAGGTTCAACATGGGATT-3’

5’-CTGAGTTGGATTCTCCCATTTT-3’

Cβ4

5’-GGAAAGGTTGGTTTTCATCATG-3’

5’-CTGAGTTGGATTCTCCCATTTT-3’

hIL-19

5’-GGCTCTGTTCCACGGGGCAT-3’

5’-AGGTGTCCTTAGCTTGGATGGCT-3’

hGCC2

5’-AAGAGACCCAGACTGTGAAG-3’

5’-TCCAGTTGCTCTGACTGCTT-3’

hPCBD2

5’-GCCATGTCATCAGGTACTCA-3’

5’-GTGGAAGGAGAATTCTTTGTAG-3’

hDicer

5’-GTCAACTCTGCAAACCAGGT-3’

5’-AATGAGAACCTGGTGCTTAG-3’

hPIP5K1a

5’-TTGTCCTCAGCATCTGGAAT-3’

5’-CAAGGCTGATGAGGTTGTCT-3’

hCBL

5’-CTCAGCCTAGGCGAAACCTA-3’

5’-CTCTTCCAAGGGACTATTGTCT-3’

hWNT9A

5’-TCCAGTTCCGCTTTGAGC-3’

5’-AGCCGAGGAGATGGCATAG-3’

hCTNNB1

5’-GCTTTCAGTTGAGCTGACCA-3’

5’-CAAGTCCAAGATCAGCAGTCTC-3’

hGAPDH

5’-TCTTTTGCGTCGCCAGCCGAGC-3’

5’-TGACCAGGCGCCCAATACGACC-3’

**shRNA pGIPZ vectors**

**(Open Biosystems)**

**shTAL1 #1** RHS4531-NM_003189 V3LHS_413459

**shTAL1 #2** RHS4531-NM_003189 V3LHS_413460

**shGATA1 #1** RHS4531_EG2623 V3LHS_248337

**shGATA1#2** RHS4531_EG2623 V3LHS_248340

**shRUNX1#1** RHS4531_NM_001001890 RHS4430-101098298 V3LHS_367629

**shRUNX1#2** RHS4531_NM_001001890 RHS4430-98894231 V2LHS_150256
